# Supplementary figures and images for: A Novel Artificial MicroRNA Expressing AAV Vector for Phospholamban Silencing in Cardiomyocytes Improves Ca2+ Uptake into the Sarcoplasmic Reticulum
Source: PLoS One. 2014 Mar 26;9(3):e92188. doi: 10.1371/journal.pone.0092188 (PMC3966758; doi:10.1371/journal.pone.0092188)

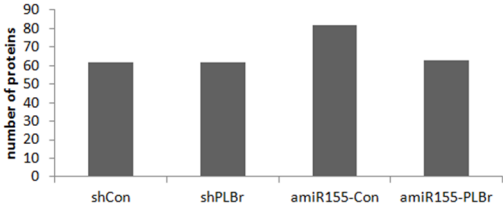

Supplement: Figure S1 — Proteins displaying alterations after shotgun proteome analysis. Number of proteins displaying alterations FC >1.5, p<0.01 at 14 days after transduction of CM with 25×103 vg/cell of scAAV6-amiR155-PLBr, scAAV6-shPLBr and respective scAAV6-amiR155-Con and scAAV6-shCon in comparison to non-transduced CM. (PDF) [file pone.0092188.s001.pdf]
