# Supplementary material for: A Novel Artificial MicroRNA Expressing AAV Vector for Phospholamban Silencing in Cardiomyocytes Improves Ca2+ Uptake into the Sarcoplasmic Reticulum
Source: PLoS One. 2014 Mar 26;9(3):e92188. doi: 10.1371/journal.pone.0092188 (PMC3966758; doi:10.1371/journal.pone.0092188)
Supplement: Figure S2 — Scatter plot of protein intensities after AAV vector transduction. Scatter plot of protein intensities of CM 14 days after transduction with 25×103 vg/cell of scAAV6-amiR155-PLBr, scAAV6-shPLBr and respective scAAV6-amiR155-Con and scAAV6-shCon expressing AAV vectors in comparison to non-transduced (no add) cells. Each dot represents one protein. Red dots represent proteins displaying significantly different levels in comparison to Mock experiment (fold change > 1.5, p<0.01). 1- PLB = phospholamban, 2- fibronectin, 3- prosaposin, 4- thrombospondin 1. (PDF) [file pone.0092188.s002.pdf]

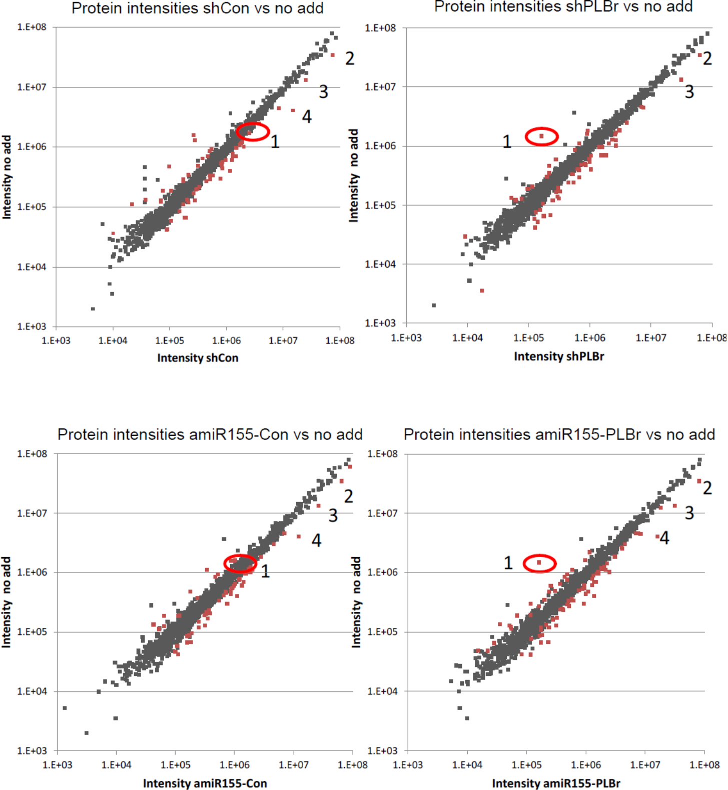
Protein intensities shCon vs no add
Intensity no add
Intensity shCon
1.E+03
1.E+04
1.E+05
1.E+06
1.E+07
1.E+08
1
2
3
4
Protein intensities shPLBr vs no add
Intensity no add
Intensity shPLBr
1
2
3
Protein intensities amiR155-Con vs no add
Intensity no add
Intensity amiR155-Con
1
2
3
4
Protein intensities amiR155-PLBr vs no add
Intensity no add
Intensity amiR155-PLBr
1
2
3
4
