# Supplementary material for: Correction: A Novel Artificial MicroRNA Expressing AAV Vector for Phospholamban Silencing in Cardiomyocytes Improves Ca2+ Uptake into the Sarcoplasmic Reticulum
Source: PLoS One. 2014 Aug 26;9(8):e107093. doi: 10.1371/journal.pone.0107093 (PMC4144974; doi:10.1371/journal.pone.0107093)
Supplement: File S2 — Republished, corrected article. (PDF) [file pone.0107093.s002.pdf]

PLOS | ONE

A Novel Artificial MicroRNA Expressing AAV Vector for Phospholamban Silencing in Cardiomyocytes Improves Ca^{2+} Uptake into the Sarcoplasmic Reticulum

Tobias Größl[1], Elke Hammer[2], Sandra Bien-Möller[3], Anja Geisler[1], Sandra Pinkert[1], Carsten Röger[1], Wolfgang Poller[4], Jens Kurreck[1], Uwe Völker[2], Roland Vetter[5]❡, Henry Fechner[1]*❡

1 Department of Applied Biochemistry, Institute of Biotechnology, Technische Universität Berlin, Berlin, Germany, **2** Interfaculty Institute for Genetics and Functional Genomics, University Medicine Greifswald, Greifswald, Germany, **3** Department of Pharmacology, Center of Drug Absorption and Transport, University Medicine Greifswald, Greifswald, Germany, **4** Department of Cardiology & Pneumology, Charité - Universitätsmedizin Berlin, Campus Benjamin Franklin, Berlin, Germany, **5** Institute of Clinical Pharmacology & Toxicology, Charité - Universitätsmedizin Berlin, Campus Charité Mitte, Berlin, Germany

Abstract

In failing rat hearts, post-transcriptonal inhibition of phospholamban (PLB) expression by AAV9 vector-mediated cardiac delivery of short hairpin RNAs directed against PLB (shPLBr) improves both impaired SERCA2a controlled Ca^{2+} cycling and contractile dysfunction. Cardiac delivery of shPLB, however, was reported to cause cardiac toxicity in canines. Thus we developed a new AAV vector, scAAV6-amiR155-PLBr, expressing a novel engineered artificial microRNA (amiR155-PLBr) directed against PLB under control of a heart-specific hybrid promoter. Its PLB silencing efficiency and safety were compared with those of an AAV vector expressing shPLBr (scAAV6-shPLBr) from an ubiquitously active U6 promoter. Investigations were carried out in cultured neonatal rat cardiomyocytes (CM) over a period of 14 days. Compared to shPLBr, amiR155-PLBr was expressed at a significantly lower level, resulting in delayed and less pronounced PLB silencing. Despite decreased knockdown efficiency of scAAV6-amiR155-PLBr, a similar increase of the SERCA2a-catalyzed Ca^{2+} uptake into sarcoplasmic reticulum (SR) vesicles was observed for both the shPLBr and amiR155-PLBr vectors. Proteomic analysis confirmed PLB silencing of both therapeutic vectors and revealed that shPLBr, but not the amiR155-PLBr vector, increased the proinflammatory proteins STAT3, STAT1 and activated STAT1 phosphorylation at the key amino acid residue Tyr701. Quantitative RT-PCR analysis detected alterations in the expression of several cardiac microRNAs after treatment of CM with scAAV6-shPLBr and scAAV6-amiR155-PLBr, as well as after treatment with its related amiR155- and shRNAs-expressing control AAV vectors. The results demonstrate that scAAV6-amiR155-PLBr is capable of enhancing the Ca^{2+} transport function of the cardiac SR PLB/SERCA2a system as efficiently as scAAV6-shPLBr while offering a superior safety profile.

Citation: Größl T, Hammer E, Bien-Möller S, Geisler A, Pinkert S, et al. (2014) A Novel Artificial MicroRNA Expressing AAV Vector for Phospholamban Silencing in Cardiomyocytes Improves Ca^{2+} Uptake into the Sarcoplasmic Reticulum. PLoS ONE 9(3): e92188. doi:10.1371/journal.pone.0092188

Editor: R. Jude Samulski, University of North Carolina at Chapel Hill, United States of America

Received September 12, 2013; **Accepted** February 19, 2014; **Published** March 26, 2014

Copyright: © 2014 Größl et al. This is an open-access article distributed under the terms of the Creative Commons Attribution License, which permits unrestricted use, distribution, and reproduction in any medium, provided the original author and source are credited.

Funding: This work was supported by the Deutsche Forschungsgemeinschaft through SFB Transregio 19 project grants to HF, UV and RV. The funders had no role in study design, data collection and analysis, decision to publish, or preparation of the manuscript.

Competing Interests: HF and JK submitted a patent application entitled "Polynucleotide sequence for the inhibition of phospholamban synthesis" (Patent application number: 20100310521, Publication date: 2010-12-09). HF and WP submitted a patent application entitled "RNA Interference for the Treatment of Heart Failure" (Patent application number: 20110098338, Publication date: 2011-04-28). This does not alter adherence to all the PLOS ONE policies on sharing data and materials, as detailed online in the guide for authors of PLOS ONE.

* E-mail: henry.fechner@tu-berlin.de

❡ These authors contributed equally to this work.

Introduction

Heart failure is the leading cause of mortality and morbidity in Western countries and a common endpoint of cardiac disorders, including atherosclerosis, ischemic cardiomyopathies, familiar cardiomyopathies, valvular-induced myocardial pathologies and arterial hypertension [1,2]. A common feature of heart failure is an altered Ca^{2+} homeostasis, which is a consequence of dysregulation of Ca^{2+} cycling proteins. The sarcoplasmatic reticulum (SR) Ca^{2+}-ATPase (SERCA2a) plays a key role in regulating normal Ca^{2+} levels in cardiomyocytes. It is the major contributor to lowering cytosolic Ca^{2+} levels and reloading the SR with Ca^{2+} during each contraction-relaxation cycle [3]. Reduced SERCA2a expression and/or activity are at least partly responsible for dysregulation of cellular Ca^{2+} homeostasis in heart failure [4], and enhancement of Ca^{2+} re-uptake activity of the cardiac SR by gene transfer of SERCA2a has been shown to improve contractile dysfunction in rodent and large animal heart failure models [5,6]. Furthermore, recent phase I and II clinical trials have confirmed the safety and clinical benefit of AAV1 vector-mediated SERCA2a gene therapy in patients with terminal heart failure [1].

The Ca^{2+} affinity and transport activity of SERCA2a are regulated by the SR protein phospholamban (PLB). Non-phosphorylated PLB keeps the Ca^{2+} affinity of SERCA2a low and reduces Ca^{2+} re-uptake into the SR, whereas PLB phosphorylation in response to β-adrenergic stimulation reverses this inhibition. The well-known regulatory role of PLB for the SERCA2a-catalyzed Ca^{2+}-transport of the cardiac SR has qualified this small membrane protein as a promising molecular target for heart failure therapy. In this context, gene therapeutic

approaches for inhibiting PLB expression by employment of dominant-negative PLB mutants [7], intracellular inhibitory antibodies targeting PLB [8] or engineered zinc-finger protein transcription factors (ZFP TFs) to the endogenous PLB gene [9] have been shown to improve the SERCA2a-catalyzed Ca^{2+} transport activity in cardiomyocytes and the contractile function of the heart in animals models of heart failure. An alternative strategy to reduce expression of PLB comprises using RNA interference (RNAi) technology [10–14]. RNAi represents a mechanism of post-transcriptional gene silencing induced by short double-stranded (ds) RNAs with length of 21-22 nucleotides [15]. The short dsRNAs are incorporated into the RNA-induced silencing complex (RISC) in the cytoplasm. One strand, the guide strand, binds to the complementary target sequence in the transcript leading to its degradation [16]. For therapeutic use, short dsRNAs can be applied as synthetic small interfering (si)RNAs, short hairpin (sh)RNAs or artificial microRNAs (amiR) [17]. After transfection, siRNAs directly target the corresponding mRNA in the cytoplasm [18]. While siRNAs are commonly used for *in vitro* investigations, their delivery for *in vivo* application is challenging with regard to target organ-specificity, transfection efficiency and long-term efficacy. An alternative is the expression of shRNAs, which contain both a sense and an antisense sequence that are connected by a loop of unpaired nucleotides. They are expressed from vectors with strong, constitutively active polymerase III promoters (U6, H1, and 7SK). After transcription, shRNAs are exported from the nucleus into the cytoplasm via the nuclear karyopherin exportin-5 and are cleaved subsequently by the RNase Dicer into functional active siRNAs [19]. The high efficiency of shRNAs in knocking down genes *in vivo* has been widely demonstrated [12,20,21]. However, shRNAs were found to induce cellular innate immune responses resulting in enhanced production of interferons (IFNs). The released IFNs can subsequently activate IFN-stimulated genes, resulting in cellular gene expression profile changes that may contribute to adverse side-effects such as global degradation of mRNA, inhibition of general protein translation or cell death [22–25]. Moreover, long-term and high-level shRNA expression can result in oversaturation of the endogenous cellular microRNA (miR) pathways leading to cytotoxicity and eventually fatality, as reported previously [26–29].

The amiRNAs represent a type of shRNAs in which a siRNA sequence is embedded into a native microRNA scaffold [30], most commonly into that of miR-30 [30,31] or miR-155 [32,33]. In contrast to shRNAs, amiRs are expressed from polymerase II promoters and are processed consecutively into functionally active siRNAs via the nuclear class 2 RNase III enzyme Drosha and the cytoplasmic endoribonuclease Dicer [34]. Several studies have compared shRNAs and amiRs regarding their efficiency and safety. The general trend of these studies is that amiRs substantially reduce the toxicity observed for the shRNAs of the same constructs [27–29]. The efficiency of gene silencing mediated by amiRNAs has been reported to be higher [27,35,36], comparable [28,37] or lower [38] in comparison to shRNAs.

We demonstrated the first successful treatment of experimental heart failure by RNAi *in vivo* [12]. An AAV9 vector and adenoviral vector-mediated cardiac expression of rat PLB-specific shRNA (shPLBr) resulted in strong cardiac down-regulation of PLB. This led to restoration of the compromised left ventricular contractile function. It also significantly reduced cardiac dilatation, hypertrophy, cardiomyocyte diameter and cardiac fibrosis. Importantly, no adverse side effects were observed using this shPLBr gene therapeutic approach in rats. More recently, however, Bish et al. [14] evaluated the efficiency and safety of an adapted shPLB in healthy dogs. They found that AAV6 vector-mediated cardiac shPLB delivery to these animals resulted in the strong knockdown of PLB, accompanied by severe cardiac toxicity.

In our present study we compared the PLB silencing efficiency and the safety profile of a newly developed AAV vector (scAAV6-amiR155-PLBr) expressing a novel engineered artificial microRNA (amiR155-PLBr) directed against PLB with those of a previously described AAV vector (scAAV6-shPLBr [12]) expressing shPLBr in neonatal rat cardiomyocytes (CM). Compared to shPLBr, amiR155-PLBr expression was delayed and lower. A comparable increase of the SERCA2a-catalyzed Ca^{2+} transport activity, however, was observed after long term treatment of CM with the respective AAV vectors. MicroRNA and proteome analysis detected similarities but also differences between amiR155-PLBr and shPLBr vectors regarding undesirable off-target effects. scAAV6-shPLBr, but not scAAV6-amiR155-PLBr, caused elevation of proinflammatory proteins such as STAT3, STAT1 and phosphorylated STAT1. Both vectors, as well as control RNAi vectors, induced alterations of microRNA expression levels in cardiac cells. Thus, scAAV6-shPLBr and scAAV6-amiR155-PLBr display comparable functional efficiency, but the safety profile of the amiR155-PLBr vector is superior to that of shPLBr vector.

Materials and Methods

Cell culture

Human embryonal kidney (HEK) 293 cells were cultured in Dulbecco's Modified Eagle's Medium (DMEM) (Life Technologies, Gibco, Darmstadt, Germany) supplemented with 10% fetal calf serum (FCS) and 1% of each penicillin/streptomycin (Sigma Aldrich Chemie GmbH, München, Germany). Primary neonatal rat CM were isolated from ventricular tissue of 1-3-day-old Wistar rat pups (Charles River, Sulzfeld, Germany) and cultured in CMRL 1415 medium (Biochrom AG, Berlin, Germany) supplemented with, 5.4 mM KCl, 1.26 mM $CaCl_2$, 10 mM Hepes, 2 µM 5′-Fluoro-2′-deoxyuridine (Sigma-Aldrich), 10% FCS and 2 µg/ml of gentamycin (Biochrom AG) as described previously [11,39]. The seeding cell density using 6-well Nunc™ Cell-Culture dishes with surface coating Nonclon Delta (Cat-No. 140685, Thermo Fisher Scientific; Rockford, IL, USA) was 1.25×10^5 cells/cm^2. Hearts were removed from decapitated rat pups and used for isolation of CM in strict accordance with the recommendations in the Guide for the Care and Use of Laboratory Animals published by the US National Institutes of Health (NIH Publication No. 85-23, revised 1996). Decapitation and removal of hearts was approved by the Landesamtes für Gesundheit und Soziales (LAGeSo) in Berlin, Germany under the Permit Number: O 0139/05.

Plasmids

Generation of amiR155 expression cassettes was performed with the Block-iT Pol II miR RNAi Expression Vector Kit according to the recommendations of the supplier (Life Technologies, Invitrogen, Darmstadt, Germany). Briefly, a double stranded (ds) oligonucleotide encoding murine miR155 pre-miRNA containing the siPLB-17 sequence [11] was generated by annealing of the primers

5′-TGCTG*TAGCCGAGCGAGTAAGGTATT*GTTTTGGCCA-CTGACTGACAATACCTTTCGCTCGGCTA-3′and

5′-CTGTAGCCGAGCGAAAGGTATTGTCAGTCAGTG-GCCAAAACAATACCTTACTCGCTCGGCTAC-3′.

The resulting ds oligonucleotide contains a TGCTG overhang at the 5′ end of the top strand and a CCTG overhang at the 5′ end of the bottom strand. The fragment was inserted into the

plasmid pcDNA6.2-GW/miR via complementary nucleotide sequences leading to the plasmid pcDNA6.2-GW/amiR155-PLBr. To generate an amiR155-PLBr expressing AAV shuttle plasmid we first digested the plasmid pscAAV-CMV-β-Intron-shPLB [12] with *Xba*I to delete the U6 promoter shPLBr expression cassette. Religation resulted in the plasmid pscAAV-CMV-β-Intron. Subsequently, the *Nde*I/*Sac*I fragment of pUF-CMV$_{enh}$/MLC0.26-Luc [40] containing the cardiac-specific CMV-enhanced 0.26 kb rat MLC promoter (CMV$_{enh}$/MLC0.26) was inserted into the *Nde*I/*Sac*I restricted pscAAV-CMV-β-Intron. The resulting plasmid was termed pscAAV-CMV$_{enh}$/MLC0.26-β-Intron. The amiR155-PLBr expression cassette was amplified by PCR using the primers misi711s-*Kpn*I 5′-CGGGGTACCCCG-GAGGTAGTGAGTCGACCAG-3′ and misi903a-*Mlu*I 5′-TCGACGCGTCGATCTCGATGCGGCCAGAT-3′ from pcDNA6.2-GW/amiR155-PLBr and inserted into the plasmid pAdR4-sCAR-Fc [41] via *Kpn*I/*Mlu*I. The resulting plasmid was digested with *Kpn*I/*Xba*I and inserted into *Kpn*I/*Xba*I digested pscAAV-CMV$_{enh}$/MLC0.26-β-Intron. The final plasmid was termed pscAAV-amiR155-PLBr.

The amiR-155 control plasmids were generated as follows. Two oligonucleotides encoding a 21 bp sequence of the RNA-dependent RNA polymerase (mature siRNA sequence: 5′-AACUUUGUUAGGUCCUUAGUC-3′) of coxsackievirus B3 (deduced from shRdRP2 sequence [20]) were annealed and inserted into pcDNA6.2-GW/miR. The resulting plasmid was termed pamiR155-Con. A 21 bp sequence (mature siRNA sequence: 5′-UAUUCAGCCCAUAUCGUUUCA-3′) of the *Firefly* luciferase (Luc) (GenBank Acc. No.: M15077) were designed with the Block-iT RNAi Designer (Life Technologies, Invitrogen) and inserted via two primer annealing into pcDNA6.2-GW/miR resulting in the plasmid pamiR155-Luc. pamiR155-Con and pamiR155-Luc were digested with *Sal*I/*Bgl*II and the miR155-Con and miR155-Luc containing fragments were inserted into *Sal*I/*Bgl*II digested pscAAV-amiR155-PLBr to replace amiR155-PLBr. The resulting plasmids were termed pscAAV-amiR155-Con and pscAAV-amiR155-Luc. To generate the plasmid pscAAV-shCon, an U6 promoter driven shCAR4m expression cassette was excised from pAd5-shCAR4m [42] with *Bam*HI/*Hind*III and inserted via *Bam*HI/*Hind*III into pscAAV-shPLBr [12]. The construct shCAR4m is directed against the murine coxsackievirus and adenovirus receptor (CAR). It does not interact with rat PLB or other proteins involved in cellular Ca^{2+} metabolism.

The reporter plasmid necessary to prove silencing activity of shPLBr and amiR155-PLBr was generated as follows. Total RNA was isolated from CM with TRIzol Reagent (Life Technologies, Invitrogen) according to the recommendation of the supplier and reverse transcribed using the High Capacity cDNA Reverse Transcription Kit (Life Technologies, Applied Biosystems Inc., Darmstadt, Germany). The cDNA of rat PLB was amplified by PCR using the primer pair *Nru*I-174s 5′-CGACTCGCGAATG-GAAAAAGTCCAATACCT-3′ and *Xba*I-332as 5′-CAGCTC-TAGAGTCACAGAAGCATCACAATGA-3′. The resulting PCR fragment containing an *Nru*I site at the 5′end and a *Xba*I site at the 3′end was inserted into the pGEM-T vector with the pGEM-T Easy Vector System II (Promega GmbH, Mannheim, Germany). The vector was digested with *Nru*I/*Xba*I and the resulting fragment was inserted into *Nru*I/*Xba*I-digested luciferase reporter plasmid pUF-CMV$_{enh}$/MLC0.26-Luc [40]. The reporter plasmid was termed pLuc-PLBr_cDNA. The correctness of the plasmids was verified by sequence analyses using an ABI 310 Genetic Analyzer (Life Technologies, Applied Biosystems Inc.).

AAV vectors

For the production of pseudotyped self-complementary (sc) AAV6 vectors HEK293 cells were co-transfected with the respective AAV shuttle plasmids pscAAV-shPLBr, pscAAV-shCon, pscAAV-amiR155-PLBr, and pscAAV-amiR155-Con and the AAV packaging plasmid pDP6 (Plasmid Factory GmbH & Co. KG, Bielefeld, Germany) providing the AAV2 *rep* genes, AAV6 *cap* genes and adenoviral helper functions. The procedure for vector generation was carried out as described previously [43]. Purification of the AAV vectors was carried out by an iodixanol step gradient centrifugation [44]. Titers of shPLBr and shCon containing AAV constructs were determined by real-time PCR using the U6 promoter specific primers 5′-AAAACTGCAAAC-TACCCAAGAAA-3′ and 5′-AAGGTCGGGCAGGAAGAG-3′ and a 6-FAM-TGCAAATATGAAGGAATCATGGGAAA-BHQ-1 labeled probe. Titers of amiR155-PLBr and amiR155-Con containing constructs were determined by real-time PCR with the primers 5′-TGCCCAGTACATGACCTTATGG-3′ and 5′-GAAATCCCCGTGAGTCAAACC-3′ and a 6-FAM-AGT-CATCGCTATTACCATGG-BHQ-1 labeled probe (Metabion GmbH, Martinsried, Germany) which were directed against a target sequence located in the CMV promoter enhancer present in the amiR155-PLBr and amiR155-Con bearing AAV vector genomes. Real Time PCR quantification was carried out with the StepOnePlus Realtime PCR System (Life Technologies, Applied Biosystems Inc.).

Plasmid transfection

HEK293 cells were seeded in 24-well plates at a density of 2×10^5 cells/well. After 24 h cells were transfected with 50 ng of the plasmid expressing *Firefly* luciferase, 75 ng of the plasmids expressing shPLBr, amiR155-PLBr, shCon, amiR155-Con and amiR155-Luc, respectively, 5 ng of a plasmid encoding *Renilla* luciferase for standardisation and 800 ng of an unrelated carrier plasmid containing a green fluorescent protein using polyethylenimine solution (Sigma-Aldrich) as transfection reagent. Analysis of reporter gene expression was carried out 72 h after transfection.

Luciferase assay

Firefly and *Renilla* luciferase activity were measured using the Dual-Luciferase Reporter Assay (Promega GmbH) according to manufacturer's instructions. Luciferase activity was measured in a Lumat LB 9507 luminometer (Berthold Technologies, Bad Wildbad, Germany).

Transduction of cardiomyocytes with AAVs

CM were seeded in 6-well- (1.2×10^6cells per well) or 24-well- (3×10^5 cells per well) cell culture plates. After two days, the medium was replaced by fresh medium and the cells were transduced with the AAV6 vectors. At this time point cells had a confluency of about 70-80%. Twenty four hours later the cell culture medium was changed and the cells were washed once with PBS and further incubated with fresh medium until the cells were harvested.

qRT-PCR

Quantification of processed shPLBr and amiR155-PLBr was carried out using two-step RT-PCR as described previously [45] with some modifications. Total RNA was isolated from CM as described and 1 µg of RNA was digested with peqGOLD DNaseI. 250 ng of DNaseI-digested RNA was reverse transcribed using the stem-loop RT primer 5′-GTCGTATCCAGTGCAGGGTCC-GAGGTATTCGCACTGGAT ACGACAATACC-3′ and the

High-Capacity cDNA Reverse Transcription Kit (Life Technologies, Applied Biosystems Inc.). Mature shPLBr and amiR155-PLBr were amplified using the primers 5′-GTGCAGGGTCC-GAGGT-3′ and 5′-GCGGCTAGCCGAGCGAGTAA-3′. For normalization of equal RNA load the expression of the 18 S rRNA was determined by quantitative RT-PCR using the primer pair 5′-CGCGGTTCTATTTTGTTGGT-3′ and 5′-AGTCGG-CATCGTTTATGGTC-3′. Real-time PCR reactions were carried out with the SYBR Green PCR Master Mix (Life Technologies, Applied Biosystems Inc.) and StepOnePlus Real-Time PCR System (Life Technologies, Applied Biosystems Inc.). Each sample was tested in triplicate.

To analyse the expression of miR-1, miR-21, miR-124, miR-195, and miR-199a, 10 ng of total RNA, isolated from CM, were reverse transcribed using the TaqMan MicroRNA Reverse Transcription Kit (Life Technologies, Applied Biosystems Inc.). Expression levels were determined by real-time PCR using the TaqMan MicroRNA Assays rno-miR1, hsa-miR21, hsa-miR124, hsa-miR195 and hsa-miR199a (Life Technologies, Applied Biosystems Inc.). For internal standardization U6snRNA expression was determined by use of TaqMan MicroRNA Assay NR_004394 (Life Technologies, Applied Biosystems Inc.).

To analyse PLBr gene expression 1 μg of total RNA was isolated from CM and digested with peqGOLD DNaseI. 250 ng of DNaseI-digested RNA were reverse transcribed using the High Capacity cDNA Reverse Transcription Kit (Life Technologies, Applied Biosystems Inc.). Expression levels were determined by real-time PCR (TaqMan Gene Expression AssayRn01434045_m1, Life Technologies, Applied Biosystems Inc.). Rat GAPDH gene expression was used for normalization (TaqMan Gene Expression Assay Rn99999616_m1, Life Technologies, Applied Biosystems Inc.).

PCR reactions for miRs and gene expression analyses were carried out in triplicate with TaqMan Universal PCR Master Mix (Life Technologies, Applied Biosystems Inc.) and the StepOnePlus Real-Time PCR System (Life Technologies, Applied Biosystems Inc.).

Northern blot analysis

Northern blot hybridization was carried out as described previously [11]. Hybridized filters were exposed to Kodak BioMax MR film (Sigma Aldrich).

Western blotting analysis

Cells were washed with PBS and solubilized in lysis buffer (20 mM Tris, pH 8.0, 10 mM NaCl, 0.5% (v/v) Triton X-100, 5 mM EDTA, 3 mM $MgCl_2$) containing a protease inhibitor mixture (Sigma-Aldrich). After boiling of the lysate at 95°C for 5 min proteins were separated on NuPAGE 4–12% Bis-Tris gels (Life Technologies, Invitrogen) under denaturing and reducing conditions and transferred onto a PVDF membrane (Bio-Rad, Hercules, CA, USA). The membranes were pre-incubated in Tris-buffered saline containing Tween 20 (TBS-T) (150 mM NaCl, 10 mM Tris-HCl, pH 8.0, 0.04% Tween-20) supplemented with 0.5% non-fat milk powder at room temperature for 1 h. Subsequently, membranes were incubated for 2 h at room temperature in TBS-T with diluted primary antibodies. Anti-PLB (1:1,000, mouse monoclonal) was from Millipore (Darmstadt, Germany) and anti-GAPDH (1:10,000, mouse monoclonal) was from Thermo Fisher Scientific Inc. (Rockford, IL, USA). Blots were washed extensively with TBS-T and probed for 1 h at room temperature with diluted (1:4,000) peroxidase-conjugated sheep anti-mouse secondary antibody (Sigma-Aldrich). Signals were visualized using Super Signal West Dura Extended Duration Substrate (Thermo Fisher Scientific Inc.). Signal intensities were quantified using FUSION FX7 Advance Analyzer and BIO-1D Advanced analysis software (PEQLAB Biotechnologie GmbH, Erlangen, Germany).

For detection of total and phosphorylated STAT1 and STAT3, 50 μg of protein lysates of three biological replicates were boiled for 3 min at 95°C, separated on a 7.5% SDS polyacrylamide gel and subsequently transferred onto a nitrocellulose transfer membrane (Schleicher&Schüll, Dassel, Germany). After blocking with 5% non-fat milk powder dissolved in TBS-T, the membranes were incubated with the following primary rabbit monoclonal antibodies at 4°C for 16 h: anti-total STAT1 (1:1,000), anti-total STAT3 (1:1,000) anti-phosphorylated STAT1-Tyr701 (1:500), anti-phosphorylated STAT3-Tyr705 (1:1,000) and anti-phosphorylated STAT3-Ser727 (1:1,000). All primary STAT antibodies were purchased from Cell Signaling Technology (Danvers, MA, USA). For normalization, we also analysed the expression of GAPDH as loading control using anti-GAPDH (1:3,000, 1 h at room temperature, mouse monoclonal, Biodesign Int., Meridian Life Science, Memphis, TN, USA). After incubation with primary antibodies blots were washed extensively with TBS-T followed by incubation with the secondary peroxidase-conjugated sheep anti-rabbit or sheep anti-mouse antibodies (both from Dako Deutschland GmbH, Hamburg, Germany) for 1 h at room temperature. Signals were visualized using ECL Western Blotting Detection Reagent (GE Healthcare Europe GmbH, Freiburg, Germany) and densitometric analysis was performed using Quantity One (Bio-Rad Laboratories GmbH, Munich, Germany). The relative optic densities of the specific bands were calculated and normalized to GAPDH.

Preparation of cardiomyocyte homogenates

At different time points after transduction of the CM, medium was removed from 6-well culture plates, the cell monolayer was washed twice with 2 ml ice-cold PBS and then removed from the bottom of each well in the presence of 0.6 ml ice-cold PBS using a plastic cell scraper. The content of three wells was transferred to a 2 ml Eppendorf tube and then sedimented by centrifugation (400 × g, 10 min, 4°C). Cell pellets were shock-frozen in liquid nitrogen and stored at -80°C until use. The pellets containing cells of three wells were homogenized in 150 μl ice-cold homogenization buffer containing 250 mM sucrose, 50 mM NaH_2PO_4, 10 mM NaF, 1 mM EDTA and 10 mM histidine, pH 7.4, 0.3 mM phenylmethylsulfonyl fluoride (PMSF) using a mini glass Teflon homogenizer on ice by 10 strokes. The three 50 μl aliquots of the homogenates obtained per pellet were immediately shock-frozen in liquid nitrogen and stored at -80°C until use. Protein was determined using the BCA™ Protein Assay (Pierce, Rockford, IL, USA) with bovine serum albumin as a standard.

Measurement of sarcoplasmic reticulum oxalate-supported Ca^{2+} uptake

Initial rates of oxalate-supported Ca^{2+} uptake into SR vesicles were estimated in homogenates of cultured neonatal rat CM by a standard procedure [39]. The reaction medium contained 40 mM imidazole (pH 7.0), 100 mM KCl, 5 mM $MgCl_2$, 5 mM Tris (hydroxymethyl)-aminomethane(Tris)-ATP, 6 mM phosphocreatine, 10 mM K-oxalate, 0.2 mM ethylene glycol-bis-(b-aminoethylether)-N,N,N′,N′-tetraacetic acid (EGTA), 10 mM NaN_3, 0.025 to 0.25 mM $^{45}CaCl_2$ (3 to 8×10^{11} Bq/mol), and 15-20 μg of homogenate protein per 0.2 ml. The resulting submicromolar and saturating free Ca^{2+} concentrations, amounting to 0.34 μM and 3.68 μM, were calculated using Fabiato's computer program as described elsewhere [46]. After 2 min of preincubation at 37°C,

the duplicate measurement was started by addition of protein. After 1, 2 and 3 min, a 50 µl sample was filtered through the 0.45 µm membrane filter ME25 (Schleicher &Schüll) using a vacuum pump. Filters were then washed twice with 3 ml ice-cold solution containing 100 mM KCl, 2 mM EGTA and 40 mM imidazole (pH 7.0). Radioactivity associated with the dry filters was determined by liquid scintillation counting. Solutions for Ca^{2+} transport measurements were made with deionizised water, p.a. (Merck, Darmstadt, Germany); contaminant Ca^{2+} did not exceed 0.005 mg/l. The $^{45}CaCl_2$ was obtained from PerkinElmer (Waltham, MA, USA). Rates of transport of Ca^{2+} into SR vesicles were calculated by linear regression of data points at 1, 2, and 3 min as described earlier [39] and measured in duplicate or triplicate. Specific Ca^{2+} uptake activity expressed in nmol Ca^{2+}/min/mg protein is defined as the rate of oxalate-supported Ca^{2+} uptake related to milligram of cell homogenate protein. Rate values obtained at the submicromolar free Ca^{2+} concentration were normalized to the respective rate values obtained at saturating free Ca^{2+} concentration as reported earlier [11]. This approach relies on the fact both the Ca^{2+} affinity and the activity of SERCA2a – at the saturating Ca^{2+} concentration - are not altered by the abundance nor the phosphorylation status of phospholamban.

Sample preparation for proteomics

At appropriate time points, cells were washed twice with 2 ml PBS and subsequently harvested with 0.2 ml of a solution containing 8 mol/l urea, 2 mol/l thiourea. The cell lysates were rapidly frozen by immersion in liquid nitrogen. Proteins were extracted by five cycles of thawing and freezing, in which the cell lysates were incubated for 10 min at 30°C with vigorous shaking at 1.400 rpm, mixed by vortexing and then frozen in liquid nitrogen again. Afterwards, high molecular nucleic acids were fragmented by sonification 3 times for 3 sec at 50% power (Sonoplus, Bandelin, Berlin, Germany). The protein extract was obtained by centrifugation for 1 h at 4°C and 16.000x*g*. The protein concentration of the supernatant was determined with a Bradford assay kit (Thermo Fisher Scientific Inc.). Sample aliquots were stored at -80°C.

Sample aliquots of 4 µg were diluted with 20 mM ammonium bicarbonate (ABC) until a final concentration of less than 1 M urea was reached. Following reduction by 2.5 mM DTT (1 h at 60°C) and alkylation by 10 mM iodoacetamide (30 min at 37°C), sequencing grade modified trypsin (Promega GmbH) at a ratio of 1:25 enzyme to protein was added and digestion proceeded for 18 h at 37°C. The reaction was stopped with a final concentration of 1% acetic acid and peptide mixtures were desalted on C-18 reverse phase material (ZipTip µ-C18, Millipore Corporation, Billerica, MA, USA). Peptides were eluted in 50% and 80% acetonitrile (ACN) each in 1% acetic acid. Pooled eluates were concentrated to 2 µl in a vacuum concentrator (5303, Eppendorf, Wesseling, Germany) and resuspended in 2% ACN in 0.1% acetic acid.

Global protein profiling by mass spectrometry and data analysis

Peptide separation was performed using a non-linear 86 min gradient of 5–40% ACN in 0.1% acetic acid at a constant flow rate of 300 nl/min on a reverse phase column (PepMap C18, 75 µm id x 15 cm, LC Packings, Idstein, Germany) operated on an Easy-nLC (Proxeon, Thermo Scientific, Dreieich, Germany). The eluate was directed into a LTQ-OrbitrapVelos mass spectrometer (ThermoElectron, Bremen, Germany) equipped with a nanoelectrospray ion source. After a survey scan in the Orbitrap analyzer, 13 high resolution MS/MS spectra were recorded in a data dependent mode with exclusion times of 60 sec.

Proteins were identified via an Elucidator web interface by an automated database search in a NCBI database (rel. 2009, limited to rat entries) using the Sequest algorithm rel. 2.7 (Sorcererbuilt 4.04, Sage-N Research Inc., Milpitas, CA, USA). Parent mass tolerance (MS) was set to 10 ppm and fragment mass tolerance 1 Da. Carbamido methylation of cysteine was a fixed modification and methionine oxidation was considered as optional modification. Peptides were annotated at a false positive rate of 1% calculated in Elucidator (Ceiba Solutions, Boston, MA, USA) based on Peptide Teller (Peptide Prophet [47]).

The obtained raw data were analyzed with the Rosetta Elucidator software version 3.3 (Ceiba Solutions) employing a workflow described earlier [48]. Briefly the following steps were carried out and realized with an in-house-created visual script (step iii to viii): (i) feature detection, (ii) feature alignment across all MS runs, (iii) filtering for features $p<0.05$, (iv) feature annotation, (v) median normalization by a feature set containing search results and therefore discriminating features arising from single-charged contaminants, (vi) combination of signal intensities of biological replicates, (vii) filtering for unique peptides, (viii) statistical analyses.

For functional classification of proteins Ingenuity Pathway Analysis, Version 8.7 (Ingenuity Systems, Redwood City, CA, USA) was used. Furthermore, this software tool allowed the identification of biological processes particularly influenced by interfering RNA approaches and reduced PLB levels.

Statistical analysis

Data are presented as means ± S.E.. For statistical comparison of Ca^{2+} transport data the Mann-Whitney Rank Sum test was used. All other tests were done using the unpaired Student's t-test. Statistical significance was assumed at $p<0.05$ or $p<0.01$ (proteome analyses).

Results

Silencing efficiency of amiR155-PLBr

To develop an improved vector system for modulation of cardiac Ca^{2+} homeostasis by silencing PLB, an siRNA sequence directed against the rat PLB [11] was embedded into the native environment of the miR-155 stem loop structure (amiR155-PLBr) and placed under control of the CMV-MLC0.26 promoter, which is specifically active in cardiac cells [43,49]. To assess its functionality, a second construct containing a previously described shPLBr under control of an ubiquitously active U6 promoter was used (**Figure 1A**). Co-transfection of HEK293 cells with either amiR155-PLBr or shPLBr expression plasmids and plasmids expressing a luciferase reporter containing the rat PLB cDNA within its 3′UTR was used to determine the PLB silencing activity of these constructs (**Figure 1A**). The control vectors expressing amiR155-Con and shCon were used to test the target specificity of the amiR155-PLBr and the shPLBr, respectively. Luciferase activity assays showed that both amiR155-PLBr and shPLBr recognized the target sequence in the PLB cDNA and efficiently silenced the luciferase reporter (**Figure 1B**).

Quantification of amiR155-PLBr and shPLBr expression

AAV vectors represent the most efficient vehicles to deliver transgenes or shRNAs to the heart. Therefore, we generated AAV vectors expressing either amiR155-PLBr (scAAV6-amiR155-PLBr), shPLBr (scAAV6-shPLBr) or the respective short regulatory control RNAs (scAAV6-shCon, scAAV6-amiR155-Con). The

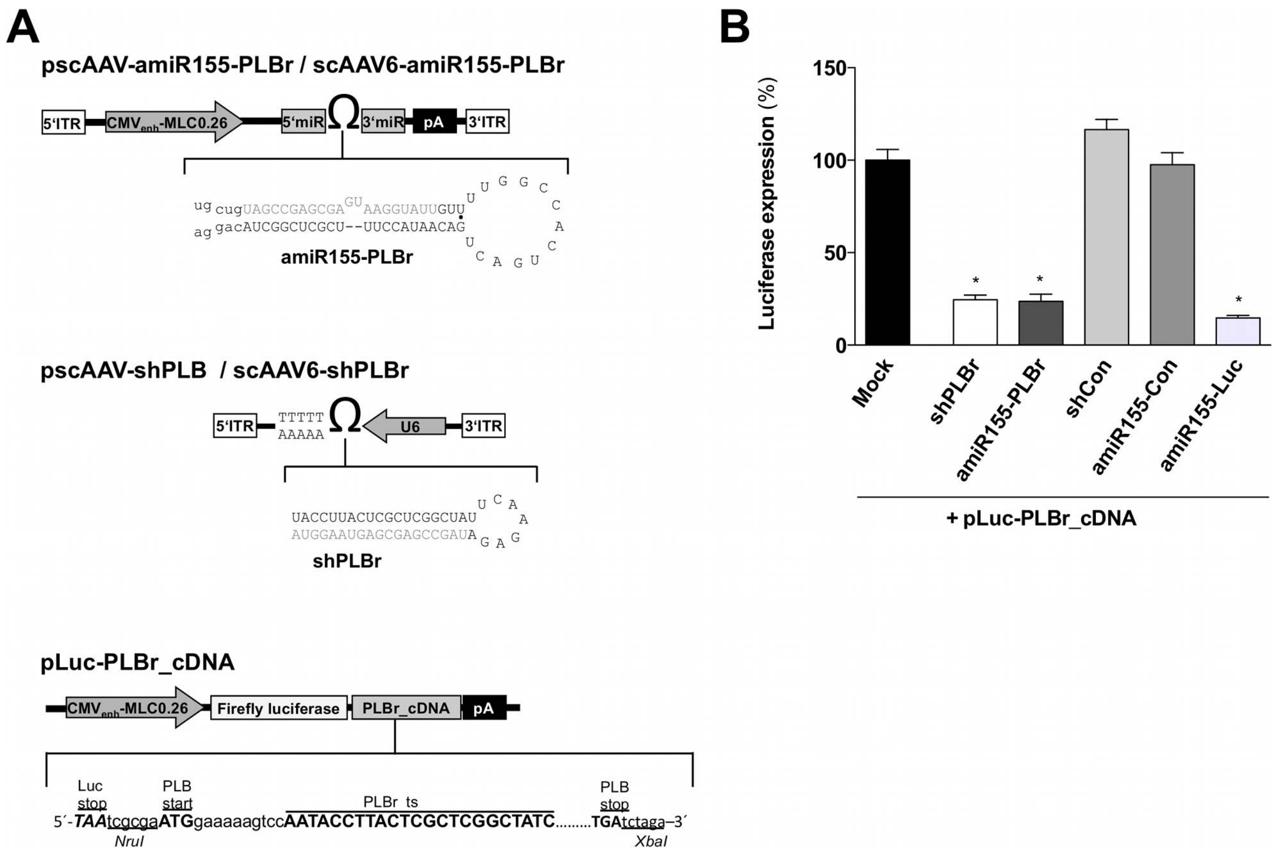

Figure 1. Scheme of miR-155-PLBr and shPLBr expressing vectors as well as of a luciferase PLB cDNA reporter gene plasmid (A) and luciferase expression in HEK293 cells co-transfected with either PLB silencing or non-silencing control plasmids (B). (A) Schematic representation of the amiR155-PLBr, shPLBr and luciferase reporter vectors. *Upper panel*: The amiR155-PLBr and shPLBr were inserted into an AAV shuttle plasmid containing an AAV2 vector backbone. The structure and nucleotide sequence of the amiR155-PLBr and shPLBr are shown below the vector scheme. The antisense strand of the mature amiR155-PLBr and shPLBr is highlighted in grey. *Middle Panel*: The shPLBr expression is driven by an U6 polymerase III promoter and terminated by five consecutive thymidines. The amiR155-PLBr expression is driven by a CMV-enhanced 0.26 kb rat MLC hybrid polymerase II promoter (CMV_{enh}-MLC0.26) and terminated by a SV40 poly A signal (pA). *Note*: The 3′ ITR of the AAV genome has a mutation in the terminal resolution site resulting in self complementary AAV vector genomes after packaging into AAV6 capsids. *Lower panel*: Schematic illustration of plasmids containing the Firefly luciferase reporter gene and the PLB cDNA (pLuc-PLBr_cDNA) in its 3′UTR. (B) Silencing activity of shPLBr and amiR155-PLBr using reporter assays. HEK293 cells were transfected with pLuc-PLBr_cDNA and *Renilla* luciferase expression plasmid and cotransfected with either amiR155-PLBr or shPLBr expressing plasmids. In addition, cotransfection was also done using the respective control plasmids amiR155-Con, shCon and amiR155-Luc. Cells were harvested 72 h after transfection and luciferase activity was determined. Firefly luciferase expression signals of the pLuc-PLB_cDNA reporter were normalized to *Renilla* luciferase and related to the respective signals in exclusively with Firefly luciferase expression vector transfected cells (Mock). *$p<0.01$ vs. respective control.
doi:10.1371/journal.pone.0092188.g001

AAV6 serotype was chosen because it has been shown to efficiently transduce cardiac cells *in vitro* [20,44]. Indeed, transduction of CM with 25×10^3 viral vector equivalents per cell (vg/c) of a GFP reporter vector resulted in GFP expression in approx. 90% of CM at post transduction day 14 (data not shown). To study possible functional differences between the used PLB silencing vectors, we first determined the expression levels of mature shPLBr and amiR155-PLBr. For this purpose, CM were transduced with scAAV6-shPLBr or scAAV6-amiR155-PLBr at doses of 0.25×10^3 to 25×10^3 vg/c. The levels of shPLBr and amiR155-PLBr were then determined by qRT-PCR at days 4 and 14 post transduction. As shown in **Figure 2A**, both shPLBr and amiR155-PLBr levels increased with rising vector doses at day 4 as well as at day 14 post transduction. The measured levels of shPLBr, however, markedly exceeded those of amiR155-PLBr, i.e. 3- to 13-fold at day 4 and 4- to 15-fold at day 14 within the tested vector dose range. These differences were inversely related to the vector-induced reduction of respective PLB mRNA levels shown in **Figure 2B**.

Dose- and time-dependent PLB silencing by amiR155-PLBr

For further detailed analysis of the influence of the investigated vectors on PLB expression at the transcriptional and translational levels, CM were transduced with either scAAV6-amiR155-PLBr, scAAV6-shPLBr or the control vector scAAV6-amiR155-Con at doses between 2.5×10^3 and 50×10^3 vg/cell. Northern blot analysis performed at day 7 post transduction revealed that a low dose of 5×10^3 vg/c scAAV6-shPLBr was sufficient to reduce PLB mRNA nearly completely (**Figure 3A**). By contrast, a much higher dose of scAAV6-amiR155-PLBr was needed to achieve the same goal (**Figure 3A**). These findings are in line with the qRT-PCR data shown in **Figure 2B**. In addition, a different vector dose-dependent decrease of immunoreactive PLB abundance was observed in CM after transduction with either scAAV6-shPLBr or

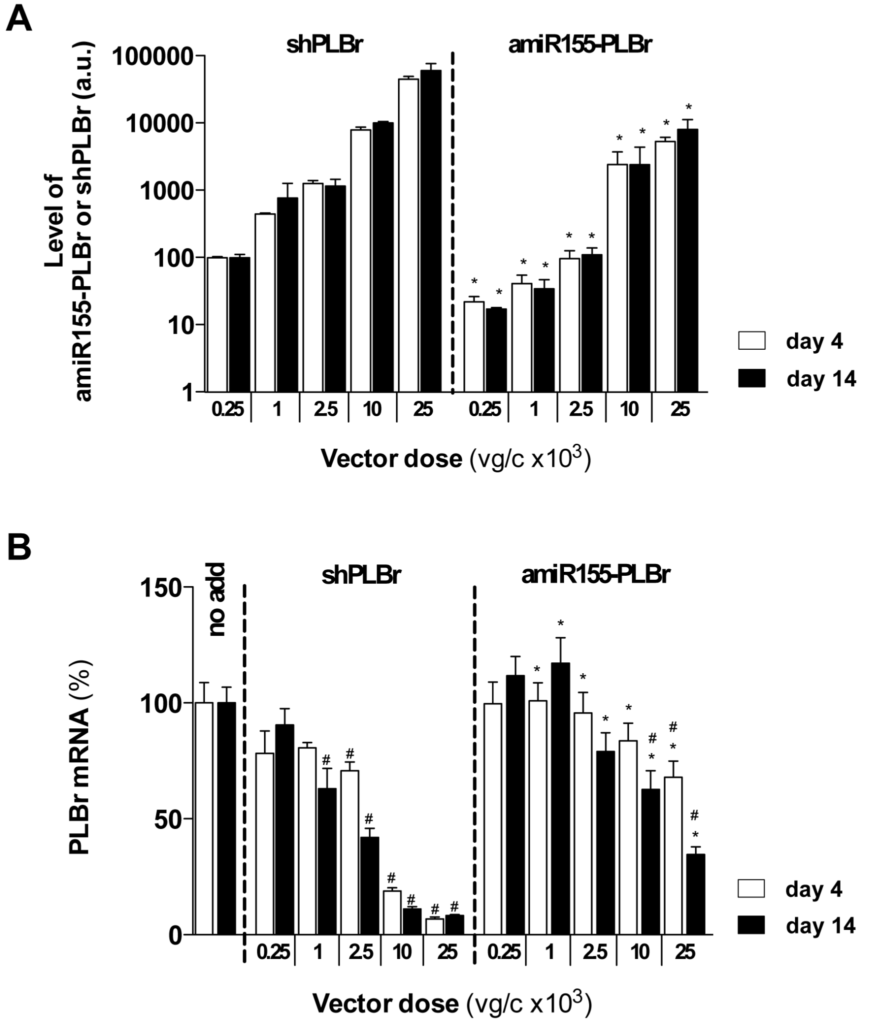

Figure 2. Vector dose dependence of amiR-155-PLBr, shPLBr and PLB mRNA expression in cardiomyocytes (CM) at post transduction days 4 and 14. CM were transduced with indicated doses of scAAV6-shPLBr and scAAV6-amiR155-PLBr and analyzed for expression of processed shPLBr and amiR155-PLBr normalized to 18 S rRNA (A) as well as PLB mRNA normalized to GAPDH mRNA using qRT-PCR (B). All values in A and B were normalized to that obtained for the cells transduced with the lowest scAAV6-shPLBr vector dose. The column labeled no add contains nontransduced cells. $^{*}p<0.05$ vs. cells transduced with the same dose of the scAAV6-shPLBr vector. $^{\#}p<0.05$ vs. non-transduced cells. a.u., arbitrary units.
doi:10.1371/journal.pone.0092188.g002

scAAV6-amiR155-PLBr (**Figure 3B, upper panel**). In fact, semi-quantitative Western blot analysis revealed a reduction of the PLB abundance to 11% of the control after transduction with 50×10^3 vg/cell of scAAV6-shPLBr, while the same dose of scAAV6-amiR155-PLBr reduced the PLB abundance to 44% of the control only (**Figure 3B, lower panel**).

We then compared the amiR155-PLBr- and shPLBr-mediated PLB silencing as a function of time after transduction. For these analyses, CM were transduced with either scAAV6-amiR155-PLBr, scAAV6-shPLBr or the control vector scAAV6-amiR155-Con using a dose of 25×10^3 vg/c of each vector. The PLB mRNA and protein levels were determined between days 1 to 14 post transduction using qRT-PCR and semiquantitative Western blot analysis (**Figure 3C-E**). At day 1 after transduction, PLB mRNA levels of all vector-treated CM did not differ from the untreated cells. After another three days, however, a strong decline of PLB mRNA to 9% of control was found in scAAV6-shPLBr treated cells. At day 14 post transduction, this value amounted to 4% of control. By contrast, a decline of the PLB mRNA levels following scAAV6-amiR155-PLBr treatment was first visible at day 7 (33% of control), and this value reached 20% of the control value at day 14 post transduction (**Figure 3C**). The quantitation of PLB protein abundance shown in **Figure 3D, E** confirms the distinct dose-dependent silencing of PLB for the two types of vectors. Thus, PLB protein levels in scAAV6-shPLBr-treated CM amounted to 14% and 2% of control at post transduction days 7 and 14, respectively. In scAAV6-amiR155-PLBr-treated cells, the respective PLB abundance was reduced to 53% and 29% of control only (**Figure 3E**). Importantly, abundance of other proteins involved in Ca^{2+} metabolism such as SERCA2a (**Figure 3D, E**), NCX1 and CSQ2 (not shown) remained unaffected in the experimental groups.

These data demonstrate that treatment of CM with scAAV6-amiR155-PLBr results in delayed and significantly reduced PLB silencing compared to treatment of the cells with its shPLB-expressing AAV6 counterpart.

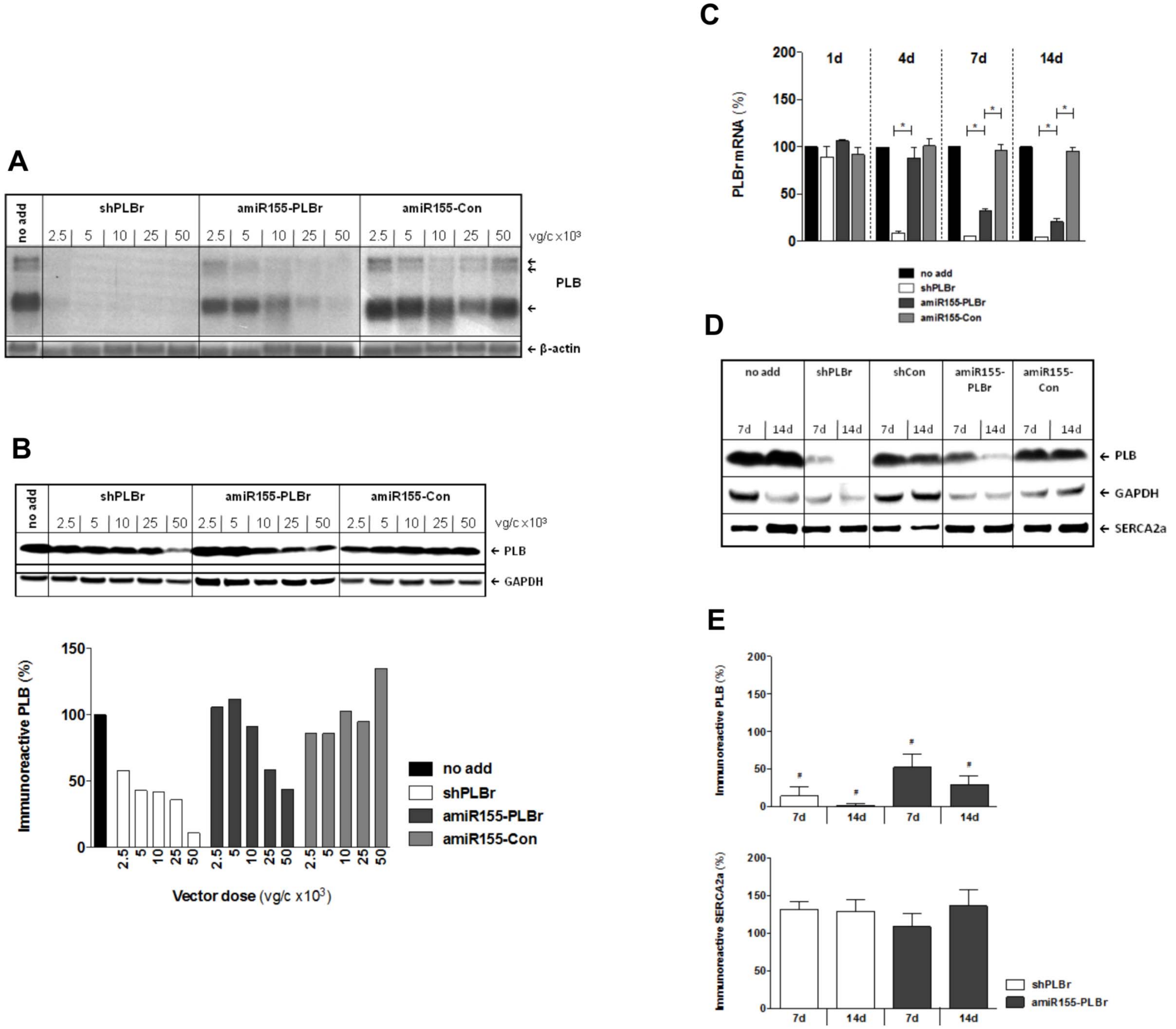

Figure 3. Silencing of PLB in CM by amiR155-PLBr and shPLBr at the mRNA and protein level. (A) Dose dependence of RNAi-mediated PLB mRNA silencing detected by Northern blot analysis. CM were transduced with either scAAV6-amiR155-PLBr (amiR155-PLBr), scAAV6-shPLBr (shPLBr) or control AAV vector (amiR155-Con) and the cells were harvested 7 days later. The multiple bands represent alternatively spliced PLB mRNA transcripts of different lengths. (B) Dose dependence of RNAi-mediated PLB silencing at the protein level for transduction conditions described under A. *Upper panel*: Representative Western blots showing the immunoreactive signals of PLB and GAPDH. *Lower panel*: Results of the semi-quantitative analysis of the blots. The obtained PLB/GAPDH signal ratio values were normalized to that of non-transduced cells (no add). (C-E) Time dependence of RNAi-mediated PLB silencing. CM were transduced with 25×10^3 vg/c of scAAV6-shPLBr, scAAV6-amiR155-PLBr, scAAV6-shCon, or scAAV6-amiR155-Con as indicated and then analyzed at indicated time points. (C) The PLB mRNA expression levels were determined by qRT-PCR. Quantification of relative PLB mRNA levels was done as described under (Figure 2B). (D) Representative Western blot of PLB, SERCA2a and GAPDH. (E) Results of the semi-quantitative Western blotting analysis of four independent experiments. The bars indicate the percentage of PLB (*upper panel*) and SERCA2a (*lower panel*) abundance found in scAAV6-shPLBr (shPLBr) and scAAV6-amiR155-PLBr (amiR155-PLBr) vector transduced cells as compared to respective control vector transduced CM exhibiting PLB and SERCA2a abundance of 100%, respectively. *$p<0.05$ shPLBr vs. amiR155-PLBr and amiR155-PLBr vs amiR155-Con; $^{\#}p<0.05$ vs. cells transduced with the respective control vector scAAV6-amiR155-Con or scAAV6-shCon.
doi:10.1371/journal.pone.0092188.g003

Changes in SR Ca^{2+} transport induced by amiR155-PLBr- and shPLBr-mediated PLB silencing

To examine functional consequences of scAAV6-amiR155-PLBr- and scAAV6-shPLBr-induced silencing of PLB on the SERCA2a-catalyzed Ca^{2+} uptake in CM, oxalate-supported SR Ca^{2+} uptake was measured in cell homogenates. In **Figure 4A**, the Ca^{2+}-dependence of the Ca^{2+} uptake rate is shown. At day 14 post transduction, there was a clear leftward shift of the sigmoidal Ca^{2+}-dependence curve due to PLB silencing by scAAV6-amiR155-PLBr. The respective EC_{50} (Ca^{2+}) value was reduced to 63% of the control value found for scAAV6-amiR155-Con-treated CM. This effect indicates increased Ca^{2+} sensitivity of the SERCA2a-mediated Ca^{2+} transport due to PLB silencing.

The stimulating effect of PLB silencing by scAAV6-amiR155-PLBr on SR Ca^{2+} uptake is clearly dependent on the time after transduction, as shown in **Figure 4B**. For the time course, SR

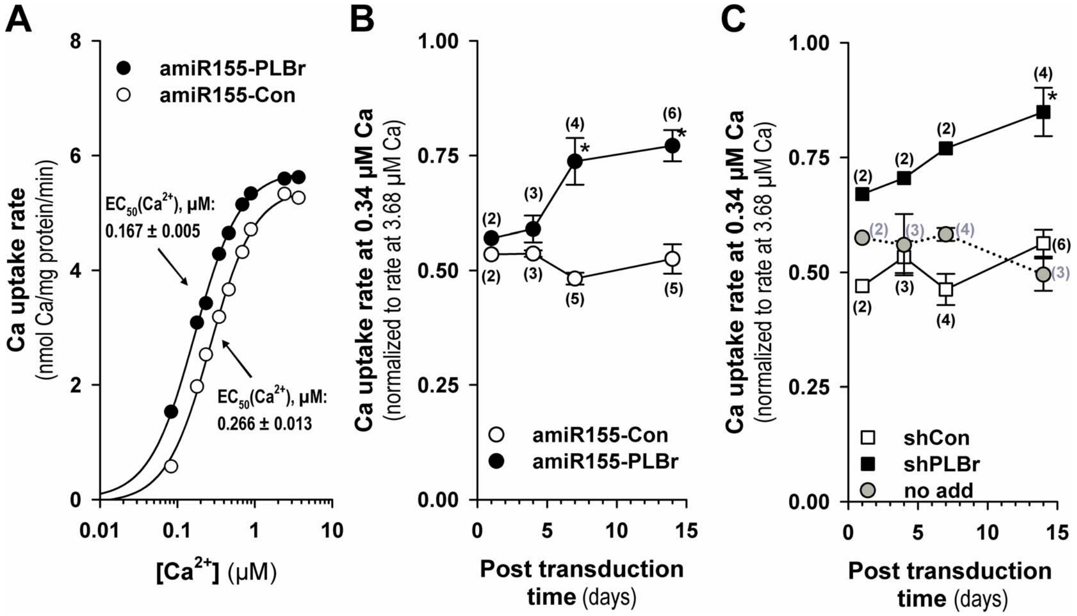

Figure 4. Sarcoplasmic reticular (SR) Ca^{2+} uptake activity in cardiomyocyte homogenates after amiR155-PLBr- and shPLBr-mediated PLB silencing. (A) Ca^{2+} dependence of the cell homogenate oxalate-supported SR Ca^{2+} uptake rate at day 14 after transduction with either scAAV6-amiR155-PLBr (amiR155-PLBr) or scAAV6-amiR155-Con (amiR155-Con) vectors. Reticular Ca^{2+} transport was measured as oxalate-facilitated Ca^{2+} uptake. Rate values are means of two different transduction experiments with primary cultured CM ($50x10^3$ vg/c). Respective rate values were obtained by linear regression analysis of Ca^{2+} uptake data obtained 1, 2, and 3 min after initiation of the reaction. (B) SR Ca^{2+} uptake rates as a function of time after transduction with $25-50\times10^3$ vg/c of either scAAV6-amiR155-PLBr (amiR155-PLBr) or scAAV6-amiR155-Con (amiR155-Con) vectors. (C) SR Ca^{2+} uptake rates as a function of time after transduction with $25-50\times10^3$ vg/c of either scAAV6-shPLBr (shPLBr) or scAAV6-shCon (shCon). For B and C, cells were harvested at indicated time points post transduction and initial oxalate-facilitated Ca^{2+} uptake rates were determined at both submicromolar (0.34 μM) and saturating (3.68 μM) free Ca^{2+} concentrations. The shown relative rate values at submicromolar Ca^{2+} in B and C were obtained by normalization of the rate value at 0.34 μM Ca^{2+} to the respective maximum uptake rate values measured at a saturating Ca^{2+} concentration of 3.68 μM. Values in B and C are means ± SEM for 2 to 6 different cell experiments as indicated in parenthesis, *$p<0.05$ vs. respective control vector. The column labeled no add contains nontransduced cells.
doi:10.1371/journal.pone.0092188.g004

Ca^{2+} uptake activity was measured at a submicromolar free Ca^{2+} concentration of 0.34 μM, at which the Ca^{2+} affinity of the Ca^{2+} transporter is known to be dependent on PLB. A significant increase in Ca^{2+} uptake activity was observed 7 and 14 days after treatment of CM with scAAV6-amiR155-PLBr. At day 14 after transduction, the normalized Ca^{2+} transport activity reached 77% of the maximal value measured at saturating Ca^{2+}. A comparable value of 85% was measured for PLB silencing with the scAAV6-shPLBr vector (**Figure 4C**). By contrast, respective relative Ca^{2+} uptake rate values around 50 to 55% of the maximum were found for CM that were treated with the two control vectors scAAV6-amiR155-Con (**Figure 4B**) and scAAV6-shCon (**Figure 4C**) as well as for non-transduced cells (**Figure 4c**) at all investigated time points. Interestingly, a detectable functional effect of PLB silencing by the shPLB-producing vector was that it exerted its functional effect on SR Ca^{2+} uptake at an earlier time point than the amiR155-PLBr producing vector (**Figure 4B, C**).

ScAAV6-shPLBr, but not scAAV6-amiR155-PLBr, induces an inflammatory response

To investigate possible side effects of both amiR155-PLBr and shPLBr-encoding vectors, the protein expression profile of CM was analyzed 14 days after transduction with $25x10^3$ vg/c of either scAAV6-amiR155-PLBr, scAAV6-shPLB, scAAV6-shCon or scAAV6-amiR155-Con. At this time point, no differences in morphology, cell growth and functionality (beating frequency) were obvious between the groups. Analysis of the 1,241 proteins covered by shotgun proteome analysis revealed that a total of 60 to 80 proteins were dysregulated through CM transduction with the AAV vectors (**Figure S1**). No major intergroup differences of the overall protein pattern were detected, except for a small subset of proteins which exhibited altered abundance levels (>1.5-fold, $p<0.05$) after transduction with scAAV6-amiR155-PLBr and scAAV6-shPLBr as compared to the respective control vectors (**Figure 5A**) or to non-transduced CM (**Figure S2**). Notably, the number of affected proteins was lower in the scAAV6-amiR155-PLBr treatment group (*vs.* scAAV6-amiR155-Con: two up- and 14 down-regulated) compared to CM transduced with scAAV6-shPLBr (*vs.* scAAV6-shCon: 12 and 18 proteins were up- and down-regulated, respectively). This analysis also confirmed efficient PLB silencing down to less than 10% of the respective control for both amiR155-PLBr and shPLBr vectors (data not shown). Furthermore, a slight increase of troponin I levels (amiR155-PLBr: FC 1.7, shPLBr: FC 1.5) was observed. Interestingly, a decreased level of the regulatory subunit of cAMP-dependent protein kinase type I (FC 1.7) was found after transduction with scAAV6-amiR155-PLBr, while the level of this protein was elevated in scAAV6-shPLBr-transduced CM. None of the other identified calcium cycling or various signaling proteins displayed altered levels after transduction with scAAV6-amiR155-PLBr. A different observation was made for CM transduced with scAAV6-shPLBr. Several proteins were altered which are involved in acute phase response signaling and inflammatory processes, such as the β-isoform of the signal transducer and activator of transcription 1 (STAT1) shown in **Figure 5B**, α-2-HS-glycoprotein, serpine peptidase inhibitor clade I, β-2-microglobulin,

calnexin and alpha-1-antitrypsin. In addition to these proteins, two IFN-regulated proteins, the galectin-3-binding protein (Gal3BP) and the major prion protein (PrP), were dysregulated by shPLBr but remained unchanged after treatment with amiR-155-PLBr. To further analyze the CM inflammatory response in more detail, Western blot analyses were performed for selected members of the JAK/STAT pathway. As shown in **Figure 5B, C**, the up-regulation of STAT1 after transduction of CM with scAAV6-shPLBr that was found by proteomics was verified by Western blot analysis (**Figure 5C**). Semiquantitative evaluation of the latter revealed an estimated 1.4-fold increase in total STAT1 abundance and a 3.3-fold rise of the level of STAT1 phosphorylated at the amino acid residue Tyr_{701} (pSTAT1Tyr701) in scAAV6-shPLBr-transduced cells as compared to CM transduced with scAAV6-shCon. In addition to STAT1, a significant 1.7-fold up-regulation of total STAT3 was seen after transduction of CM with scAAV6-shPLBr compared to scAAV6-shCon. In contrast to STAT1, however, the phosphorylation status of STAT3 was not altered at the time point measured. Most notably, neither the total nor the phosphorylated levels of STAT1 or STAT3 were altered after transduction of CM with the amiR155-PLBr encoding vector (**Figure 5B, C**).

Alteration of cardiac miR levels by scAAV6- amiR155-PLBr or scAAV6-shPLBr

Bish et al. [14] reported that strong cardiac shPLB expression in canines altered the expression of several miRs and induced toxic side effects such as transiently increased serum levels of troponin I. To investigate whether scAAV6-shPLBr and scAAV6-amiR155-PLBr treatment might affect the expression levels of selected cardiac miRs that had been analyzed in the *in vivo* study of Bish et al. [14], the levels of miR-1, miR-21, miR-124, miR-195 and miR-199a were determined in CM at day 14 after transduction with 25×10^3 vg/c of scAAV6-shPLBr, scAAV6-amiR155-PLBr, scAAV6-shCon or scAAV6-amiR155-Con. We detected a common decrease of the miRs expression levels between 20–50% for all vectors compared to non-transduced control CM. Interestingly, among the investigated miRs, the miR-21 expression levels were significantly increased by scAAV6-shPLBr and scAAV6-amiR155-PLBr compared to its respective shCon and amiR155-Con control vectors. No such changes were found for any of the other quantified miR (**Figure 6**).

Discussion

Here, we show that scAAV6-amiR155-PLBr, which expresses a newly engineered small regulatory RNA directed against the negative SERCA2a modulatory protein PLB, improves the SERCA2a-catalyzed Ca^{2+} transport activity of the SR in CM. The efficiency of scAAV6-amiR155-PLBr was as high as that of an AAV6 vector expressing the conventional shPLBr that was previously used for PLB silencing [11,12]. Importantly, our data reveal that scAAV6-amiR155-PLBr exhibits an improved cardiac safety compared to the shPLBr-expressing vector.

In a previous study, we demonstrated the therapeutic efficacy of shPLBr in a rat heart failure model using AAV9 vectors for cardiac delivery. No negative side effects were detected in this study [12]. Severe cardiac toxicity, however, was observed in healthy canines after AAV6 vector-mediated delivery of shPLB [14]. Reducing the expression of the shRNA by decreasing the vector dose or the use of weaker promoters are potential ways to prevent shRNA-induced side effects [26,50]. This procedure, however, is not always successful and shRNAs can remain toxic, even when shRNA levels are reduced [28]. Moreover, shRNA expression levels below a critical value can lead to markedly reduced silencing effect [28]. Thus, reduction of shRNA toxicity might come at the price of loss of therapeutic efficiency.

A new class of engineered regulatory RNAs, amiRs, appears to be safer than shRNAs [27–29]. For this reason we have used and characterized amiR155-PLBr for silencing of PLB in CM. In accordance with previous reports [27,28,51] comparing shRNAs versus amiRs approaches, we found that amiR155-PLBr was expressed at a lower level (about 10-fold) than shPLBr. The lower activity of the heart-specific CMV-MLC0.26 promoter [43] driving the expression of miR155-PLBr compared to the highly active U6 promoter used for shPLBr delivery is the most reasonable explanation for the observed differences between the two expression systems. We cannot rule out, however, that other factors, for example individual structural characteristics, cause the differential expression of amiR155-PLBr and shPLBr. The lower expression of amiR155-PLBr was linked to distinctly (about 10-fold) less pronounced PLB silencing. Interestingly, when both amiR155-PLBr and shPLBr were expressed at similar levels, achieved by about 10-fold increase of scAAV6-amiR155-PLBr vector dose, PLB silencing was similar (**Figure. 2A and B**). Thus, our data suggest, that amiR155-PLBr and shPLBr have *per se* similar silencing efficiency. This, however, is somewhat contradictory to previous reports. McBride et al. [28] and Mazcuga et al. [51] found comparable silencing efficiency of amiRs and shRNAs despite the lower expression they found for amiRs. Other studies confirmed these data and reported higher efficacy of amiRs as compared to shRNAs [27,35,36,51]. So far, the mechanisms underlying the differing activities of amiRs and shRNA are not well understood. Differences in stability [28] and processing to generate mature siRNA molecules [37] may be important factors. In particular, the specific structure of amiRs mimicking the naturally occurring cellular miR structure has been assumed to be a determinant that makes amiRs more efficient than shRNAs [28]. In addition, the use of different termination signals and the distance between the promoter, the shRNA/amiR sequence and the termination signal may affect the respective silencing efficiency [37,51]. Although no in-depth analysis has been carried out yet, sequence-specific determinants may play an important role as well. This would explain why, in some cases, amiRs are more efficient than shRNAs, while in other cases, amiRs and shRNAs have similar activity or amiRs may even be less efficient. We confirmed that the silencing efficiency of amiR155-PLBr used here was comparable to that using a miR-30 scaffold (data not shown). Hence, the miR-155 scaffold does not affect the inhibitory activity of amiRs.

The repression of the PLB protein in CM by amiR155-PLBr by 50% and 70% at days 7 and 14 post transduction, respectively, was considerably lower than that induced by shPLBr (decline of 85% at day 7 and $>$ 95% at day 14). Nevertheless, the amiR155-PLBr-mediated PLB silencing was functionally as efficient as that of shPLBr, i.e. the increase of SERCA2a-mediated SR Ca^{2+} transport was similar for both small regulatory RNAs. Hence, the diminished PLB silencing by amiR155-PLBr appears to be functionally irrelevant in the case studied here. The effect observed here may be explained by a functional threshold for PLB-induced inhibition of SERCA2a. Both silencing strategies (amiR155-PLBr and shPLBr) diminished PLB to a level below this value. Stronger PLB silencing will thus not result in further enhancement of SERCA2a-mediated SR Ca^{2+} transport.

Avoiding undesirable side effects is critical for therapeutic applications since they restrict application of the therapeutic agent in humans. Thus, we have carried out a proteomic analysis and validated some of our findings by semiquantitative Western blot

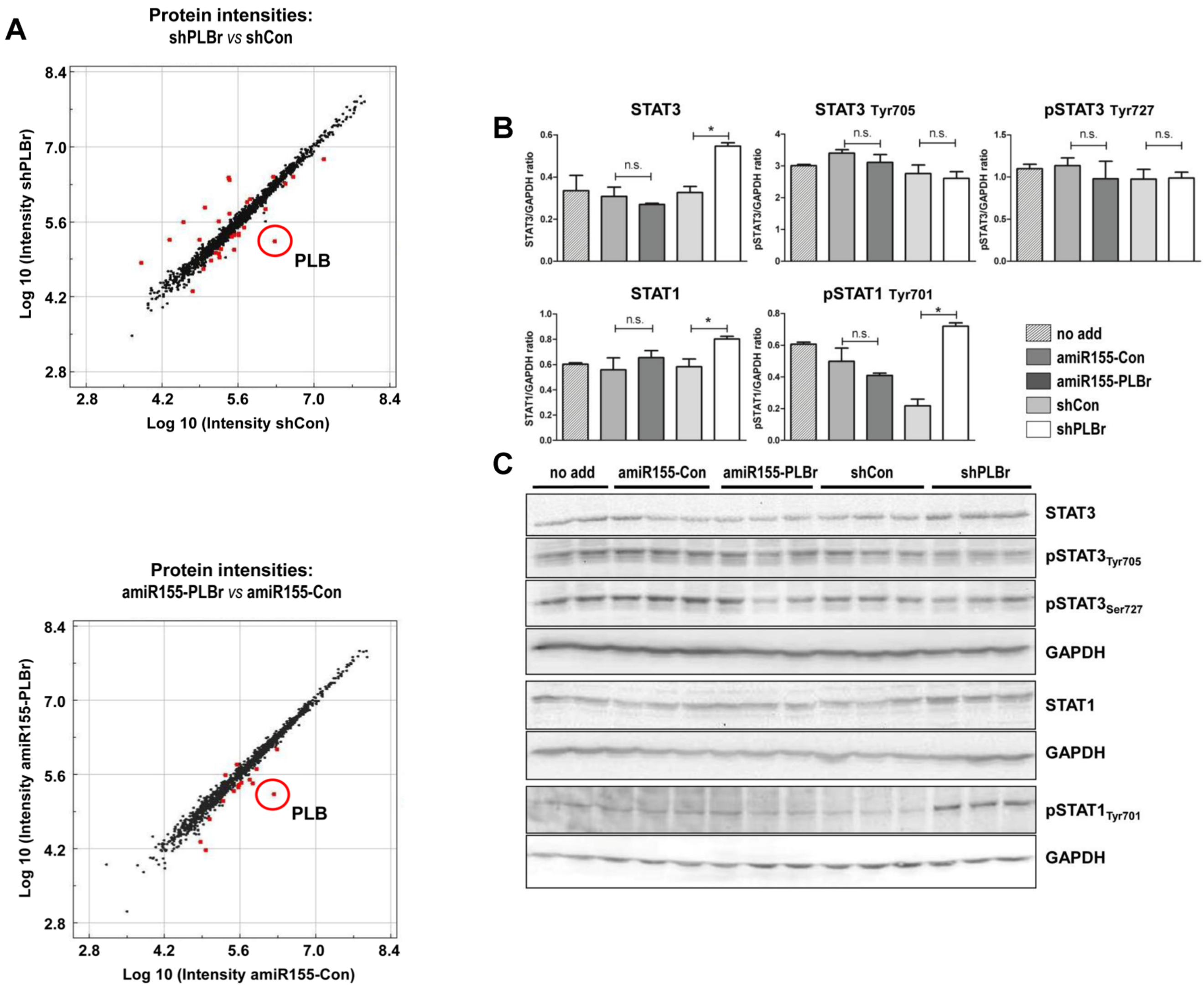

Figure 5. Alterations in the global protein pattern in scAAV6-amiR155-PLBr and scAAV6-shPLB transduced cardiomyocytes assessed by shot gun proteomics and validation by Western Blot. (A) Scatter plot of protein intensities of cardiomyocytes (CM) 14 days after transduction with 25×10^3 vg/c of scAAV6-shPLBr (shPLBr), scAAV6-amiR155-PLBr (amiR155-PLBr) or the control vectors scAAV6-shCon (shCon) and scAAV6-amiR155-Con (amiR155-Con). Each dot represents one protein. Red dots represent proteins displaying significantly different levels in comparison to control experiment (fold change >1.5, p<0.01). PLB is phospholamban protein. (B and C) Western Blot analysis of STAT1 and STAT3. CM were transduced and analyzed at 14 days after transduction as in A. (B) Quantitative analysis of three bioreplicates per assay. STAT-specific signal intensities were normalized to GAPDH as loading control. (C) Western Blot images of total and phosphorylated STAT1 and STAT3 were analyzed on 3 separate membranes. GAPDH was detected as a house keeping protein on each membrane. *p<0.05 significant intergroup difference as indicated; n.s., not significantly different.
doi:10.1371/journal.pone.0092188.g005

analysis. Importantly, shPLBr, but not amiR155-PLBr, induced the expression of the IFN-regulated proteins PrP and Gal3BP and the proinflammatory genes STAT1 and STAT3, accompanied by STAT1 activation via phosphorylation at the Tyr_{701} residue. Both STAT proteins are part of the Janus kinase (JAK)/STAT pathway, which mediates transduction of stress signals from the plasma membrane to the nucleus. IFNs are important triggers of the JAK/STAT pathway and its activation has been described in association with shRNA treatment [22,24]. Hence, our results demonstrate that long-term and high level shPLBr expression can induce undesirable induction of stress response genes in CM, probably via IFN-dependent pathways. This activation may be avoided by the use of amiR155-PLB. We also found dysregulation of several other proteins after shPLBr and amiR155-PLBr vector treatment. In most cases this may be caused by adaptation of the cellular protein network to the changed cellular metabolism as a result of PLB downregulation. Whether these findings will have relevance for the *in vivo* application of the vectors is currently not clear. It should, however, be considered in follow up studies.

We also detected alterations in the expression of cardiac miRs after transduction of CM with the shRNA and amiR155 expressing AAV vectors used in this study. Compared to untreated CM, the miRs levels were distinctly reduced, indicating that RNAi vectors have affected the miR biosynthesis. Importantly, miR downregulation occurred through treatment with PLB-silencing as well as its respective shRNA and amiR155 expressing control vectors. Hence, the suppressing effect was not restricted to shPLBr and amiR155-PLBr but represented a common outcome of AAV

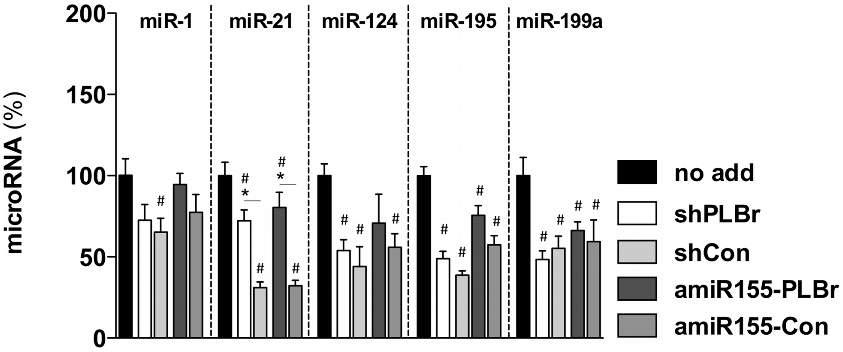

Figure 6. Alteration of miR expression in scAAV6-amiR155-PLBr and scAAV6-shPLB transduced CM. Cells were transduced with 25×10^3 vg/c of either scAAV6-shPLBr (shPLBr), scAAV6-amiR155-PLBr (amiR155-PLBr) or control vectors (scAAV6-shCon (shCon) or scAAV6-amiR155-Con (amiR155-Con)). Cells were harvested at day 14 post transduction and qRT-PCR analysis was carried out. Expression values were normalized to the level of the U6snRNA and related to that obtained for untransduced cells; $^{\#}p<0.05$ vs. untransduced cells (no add); $^{*}p<0.05$ vs. respective control vectors.
doi:10.1371/journal.pone.0092188.g006

vector-mediated transduction and expression of interfering RNAs. High shRNA expression can interfere with the cellular miR expression [14,26] and thus high vector doses used here, leading to strong shRNAs and amiR155 expression, may be the reason for our observations. Vector transduction effects, however, have also been taken into account. In a recent study, Bish et al. [14] detected dysregulation of several cardiac miR after AAV6-shPLB application to dogs. In contrast to our observations, they detected down- and up-regulation of miRs. The state of cellular differentiation (neonatal cardiomyocytes vs. adult cardiomyocytes), species-specific differences as well as differences between the *in vitro* and *in vivo* situation may be possible explanations for these differences. In this regard it should also be noted that very strong downregulation of PLB as triggered by RNAi-inducing vectors may be rather undesirable for treatment of heart failure in humans, because in humans PLB is essential for cardiac health and its absence results in lethal heart failure [52]. Thus it is obvious that further improvement of the safety of the RNAi vectors is desirable. An increase of safety might be accomplished by the use of gene expression systems subject to pharmacological regulation. For example, the tetracycline-dependent gene expression system that has already been used successfully for the expression of amiR [53] may be easily adaptable for expression of amiR155-PLBr. The system allows adjustment amiR expression within a therapeutic window, as well as outright on-off switching.

Another interesting finding of the miR analysis was a significantly higher expression of miR-21 in CM after treatment with PLB silencing vectors as compared to the respective control vectors. MiR-21 is overexpressed in many tumors and considered as key regulator of oncogenic processes [54]. It is also highly expressed in CM [55] and cardiac fibroblasts [56] and apparently plays an important role in cardiac metabolism. Both miR-21 regulation as well as PLB-SERCA2a interaction are known to be regulated via β-adrenergic receptor signaling [57,58]. Thus our data suggest that PLB-SERCA2a system could be an upstream regulator of miR-21 in the β-adrenergic signaling cascade.

In conclusion, our data suggest that the amiR155-PLB AAV vector could be a superior new RNAi vector system for improving the compromised Ca^{2+} handling function of the cardiac SR. The efficiency and the safety of this novel tool will have to be tested in experimental heart failure animal models in further studies.

Supporting Information

Figure S1 Proteins displaying alterations after shotgun proteome analysis. Number of proteins displaying alterations FC >1.5, $p<0.01$ at 14 days after transduction of CM with 25×10^3 vg/cell of scAAV6-amiR155-PLBr, scAAV6-shPLBr and respective scAAV6-amiR155-Con and scAAV6-shCon in comparison to non-transduced CM.
(PDF)

Figure S2 Scatter plot of protein intensities after AAV vector transduction. Scatter plot of protein intensities of CM 14 days after transduction with 25×10^3 vg/cell of scAAV6-amiR155-PLBr, scAAV6-shPLBr and respective scAAV6-amiR155-Con and scAAV6-shCon expressing AAV vectors in comparison to non-transduced (no add) cells. Each dot represents one protein. Red dots represent proteins displaying significantly different levels in comparison to Mock experiment (fold change $>$ 1.5, $p<0.01$). 1- PLB = phospholamban, 2- fibronectin, 3- prosaposin, 4- thrombospondin 1.
(PDF)

Acknowledgments

The technical assistance of Petra Pietsch is gratefully acknowledged. We also thank Erik Wade for a critical reading of the manuscript.

Author Contributions

Conceived and designed the experiments: TG EH SBM JK RV HF. Performed the experiments: TG EH SBM SP CR AG RV. Analyzed the data: TG EH SBM RV HF. Contributed reagents/materials/analysis tools: EH SBM WP JK UV RV HF. Wrote the paper: TB JK EH RV HF.

References

1. Hulot JS, Senyei G, Hajjar RJ (2012) Sarcoplasmic reticulum and calcium cycling targeting by gene therapy. Gene Ther 19: 596–599.
2. Lompre AM, Hajjar RJ, Harding SE, Kranias EG, Lohse MJ, et al. (2010) Ca2+ cycling and new therapeutic approaches for heart failure. Circulation 121: 822–830.
3. Hadri L, Hajjar RJ (2011) Calcium cycling proteins and their association with heart failure. Clin Pharmacol Ther 90: 620–624.
4. Kawase Y, Ladage D, Hajjar RJ (2011) Rescuing the failing heart by targeted gene transfer. J Am Coll Cardiol 57: 1169–1180.

5. del Monte F, Williams E, Lebeche D, Schmidt U, Rosenzweig A, et al. (2001) Improvement in survival and cardiac metabolism after gene transfer of sarcoplasmic reticulum Ca(2+)−ATPase in a rat model of heart failure. Circulation 104: 1424–1429.
6. Kawase Y, Ly HQ, Prunier F, Lebeche D, Shi Y, et al. (2008) Reversal of cardiac dysfunction after long-term expression of SERCA2a by gene transfer in a pre-clinical model of heart failure. J Am Coll Cardiol 51: 1112–1119.
7. Iwanaga Y, Hoshijima M, Gu Y, Iwatate M, Dieterle T, et al. (2004) Chronic phospholamban inhibition prevents progressive cardiac dysfunction and pathological remodeling after infarction in rats. J Clin Invest 113: 727–736.
8. Dieterle T, Meyer M, Gu Y, Belke DD, Swanson E, et al. (2005) Gene transfer of a phospholamban-targeted antibody improves calcium handling and cardiac function in heart failure. Cardiovasc Res 67: 678–688.
9. Zhang HS, Liu D, Huang Y, Schmidt S, Hickey R, et al. (2012) A designed zinc-finger transcriptional repressor of phospholamban improves function of the failing heart. Mol Ther 20: 1508–1515.
10. Watanabe A, Arai M, Yamazaki M, Koitabashi N, Wuytack F, et al. (2004) Phospholamban ablation by RNA interference increases Ca2+ uptake into rat cardiac myocyte sarcoplasmic reticulum. J Mol Cell Cardiol 37: 691–698.
11. Fechner H, Suckau L, Kurreck J, Sipo I, Wang X, et al. (2007) Highly efficient and specific modulation of cardiac calcium homeostasis by adenovector-derived short hairpin RNA targeting phospholamban. Gene Ther 14: 211–218.
12. Suckau L, Fechner H, Chemaly E, Krohn S, Hadri L, et al. (2009) Long-term cardiac-targeted RNA interference for the treatment of heart failure restores cardiac function and reduces pathological hypertrophy. Circulation 119: 1241–1252.
13. Andino LM, Takeda M, Kasahara H, Jakymiw A, Byrne BJ, et al. (2008) AAV-mediated knockdown of phospholamban leads to improved contractility and calcium handling in cardiomyocytes. J Gene Med 10: 132–142.
14. Bish LT, Sleeper MM, Reynolds C, Gazzara J, Withnall E, et al. (2011) Cardiac gene transfer of short hairpin RNA directed against phospholamban effectively knocks down gene expression but causes cellular toxicity in canines. Hum Gene Ther 22: 969–977.
15. Elbashir SM, Lendeckel W, Tuschl T (2001) RNA interference is mediated by 21- and 22-nucleotide RNAs. Genes Dev 15: 188–200.
16. Lee YS, Nakahara K, Pham JW, Kim K, He Z, et al. (2004) Distinct roles for Drosophila Dicer-1 and Dicer-2 in the siRNA/miRNA silencing pathways. Cell 117: 69–81.
17. Fechner H, Pinkert S, Geisler A, Poller W, Kurreck J (2011) Pharmacological and biological antiviral therapeutics for cardiac coxsackievirus infections. Molecules 16: 8475–8503.
18. Kurreck J (2009) RNA interference: from basic research to therapeutic applications. Angew Chem Int Ed Engl 48: 1378–1398.
19. Brummelkamp TR, Bernards R, Agami R (2002) A system for stable expression of short interfering RNAs in mammalian cells. Science 296: 550–553.
20. Fechner H, Sipo I, Westermann D, Pinkert S, Wang X, et al. (2008) Cardiac-targeted RNA interference mediated by an AAV9 vector improves cardiac function in coxsackievirus B3 cardiomyopathy. J Mol Med (Berl) 86: 987–997.
21. Huang Z, Dong L, Chen J, Gao F, Zhang Z, et al. (2012) Low-molecular weight chitosan/vascular endothelial growth factor short hairpin RNA for the treatment of hepatocellular carcinoma. Life Sci 91: 1207–1215.
22. Bridge AJ, Pebernard S, Ducraux A, Nicoulaz AL, Iggo R (2003) Induction of an interferon response by RNAi vectors in mammalian cells. Nat Genet 34: 263–264.
23. Hutson TH, Foster E, Dawes JM, Hindges R, Yanez-Munoz RJ, et al. (2012) Lentiviral vectors encoding short hairpin RNAs efficiently transduce and knockdown LINGO-1 but induce an interferon response and cytotoxicity in central nervous system neurones. J Gene Med 14: 299–315.
24. Kenworthy R, Lambert D, Yang F, Wang N, Chen Z, et al. (2009) Short-hairpin RNAs delivered by lentiviral vector transduction trigger RIG-I-mediated IFN activation. Nucleic Acids Res 37: 6587–6599.
25. Singh S, Narang AS, Mahato RI (2011) Subcellular fate and off-target effects of siRNA, shRNA, and miRNA. Pharm Res 28: 2996–3015.
26. Grimm D, Streetz KL, Jopling CL, Storm TA, Pandey K, et al. (2006) Fatality in mice due to oversaturation of cellular microRNA/short hairpin RNA pathways. Nature 441: 537–541.
27. Borel F, van Logtenstein R, Koornneef A, Maczuga P, Ritsema T, et al. (2011) In vivo knock-down of multidrug resistance transporters ABCC1 and ABCC2 by AAV-delivered shRNAs and by artificial miRNAs. J RNAi Gene Silencing 7: 434–442.
28. McBride JL, Boudreau RL, Harper SQ, Staber PD, Monteys AM, et al. (2008) Artificial miRNAs mitigate shRNA-mediated toxicity in the brain: implications for the therapeutic development of RNAi. Proc Natl Acad Sci U S A 105: 5868–5873.
29. Boudreau RL, Martins I, Davidson BL (2009) Artificial microRNAs as siRNA shuttles: improved safety as compared to shRNAs in vitro and in vivo. Mol Ther 17: 169–175.
30. Zeng Y, Wagner EJ, Cullen BR (2002) Both natural and designed micro RNAs can inhibit the expression of cognate mRNAs when expressed in human cells. Mol Cell 9: 1327–1333.
31. Dickins RA, Hemann MT, Zilfou JT, Simpson DR, Ibarra I, et al. (2005) Probing tumor phenotypes using stable and regulated synthetic microRNA precursors. Nat Genet 37: 1289–1295.
32. Xiao S, Wang Q, Gao J, Wang L, He Z, et al. (2011) Inhibition of highly pathogenic PRRSV replication in MARC-145 cells by artificial microRNAs. Virol J 8: 491.
33. Chung KH, Hart CC, Al-Bassam S, Avery A, Taylor J, et al. (2006) Polycistronic RNA polymerase II expression vectors for RNA interference based on BIC/miR-155. Nucleic Acids Res 34: e53.
34. Denli AM, Tops BB, Plasterk RH, Ketting RF, Hannon GJ (2004) Processing of primary microRNAs by the Microprocessor complex. Nature 432: 231–235.
35. Shan Z, Lin Q, Deng C, Li X, Huang W, et al. (2009) An efficient method to enhance gene silencing by using precursor microRNA designed small hairpin RNAs. Mol Biol Rep 36: 1483–1489.
36. Boden D, Pusch O, Silbermann R, Lee F, Tucker L, et al. (2004) Enhanced gene silencing of HIV-1 specific siRNA using microRNA designed hairpins. Nucleic Acids Res 32: 1154–1158.
37. Maczuga P, Lubelski J, van Logtenstein R, Borel F, Blits B, et al. (2012) Embedding siRNA sequences targeting Apolipoprotein B100 in shRNA and miRNA scaffolds results in differential processing and in vivo efficacy. Mol Ther.
38. Boudreau RL, Monteys AM, Davidson BL (2008) Minimizing variables among hairpin-based RNAi vectors reveals the potency of shRNAs. RNA 14: 1834–1844.
39. Vetter R, Kott M, Schulze W, Rupp H (1998) Influence of different culture conditions on sarcoplasmic reticular calcium transport in isolated neonatal rat cardiomyocytes. Mol Cell Biochem 188: 177–185.
40. Muller OJ, Schinkel S, Kleinschmidt JA, Katus HA, Bekeredjian R (2008) Augmentation of AAV-mediated cardiac gene transfer after systemic administration in adult rats. Gene Ther 15: 1558–1565.
41. Pinkert S, Westermann D, Wang X, Klingel K, Dorner A, et al. (2009) Prevention of cardiac dysfunction in acute coxsackievirus B3 cardiomyopathy by inducible expression of a soluble coxsackievirus-adenovirus receptor. Circulation 120: 2358–2366.
42. Fechner H, Pinkert S, Wang X, Sipo I, Suckau L, et al. (2007) Coxsackievirus B3 and adenovirus infections of cardiac cells are efficiently inhibited by vector-mediated RNA interference targeting their common receptor. Gene Ther 14: 960–971.
43. Geisler A, Jungmann A, Kurreck J, Poller W, Katus HA, et al. (2011) microRNA122-regulated transgene expression increases specificity of cardiac gene transfer upon intravenous delivery of AAV9 vectors. Gene Ther 18: 199–209.
44. Sipo I, Fechner H, Pinkert S, Suckau L, Wang X, et al. (2007) Differential internalization and nuclear uncoating of self-complementary adeno-associated virus pseudotype vectors as determinants of cardiac cell transduction. Gene Ther 14: 1319–1329.
45. Varkonyi-Gasic E, Hellens RP (2010) qRT-PCR of Small RNAs. Methods Mol Biol 631: 109–122.
46. Cernohorsky J, Kolar F, Pelouch V, Korecky B, Vetter R (1998) Thyroid control of sarcolemmal Na+/Ca2+ exchanger and SR Ca2+-ATPase in developing rat heart. Am J Physiol 275: H264–273.
47. Keller A, Nesvizhskii AI, Kolker E, Aebersold R (2002) Empirical statistical model to estimate the accuracy of peptide identifications made by MS/MS and database search. Anal Chem 74: 5383–5392.
48. Hammer E, Goritzka M, Ameling S, Darm K, Steil L, et al. (2011) Characterization of the human myocardial proteome in inflammatory dilated cardiomyopathy by label-free quantitative shotgun proteomics of heart biopsies. J Proteome Res 10: 2161–2171.
49. Muller OJ, Leuchs B, Pleger ST, Grimm D, Franz WM, et al. (2006) Improved cardiac gene transfer by transcriptional and transductional targeting of adeno-associated viral vectors. Cardiovasc Res 70: 70–78.
50. Giering JC, Grimm D, Storm TA, Kay MA (2008) Expression of shRNA from a tissue-specific pol II promoter is an effective and safe RNAi therapeutic. Mol Ther 16: 1630–1636.
51. Maczuga P, Koornneef A, Borel F, Petry H, van Deventer S, et al. (2012) Optimization and comparison of knockdown efficacy between polymerase II expressed shRNA and artificial miRNA targeting luciferase and Apolipoprotein B100. BMC Biotechnol 12: 42.
52. Haghighi K, Kolokathis F, Pater L, Lynch RA, Asahi M, et al. (2003) Human phospholamban null results in lethal dilated cardiomyopathy revealing a critical difference between mouse and human. J Clin Invest 111: 869–876.
53. Shin KJ, Wall EA, Zavzavadjian JR, Santat LA, Liu J, et al. (2006) A single lentiviral vector platform for microRNA-based conditional RNA interference and coordinated transgene expression. Proc Natl Acad Sci U S A 103: 13759–13764.
54. Selcuklu SD, Donoghue MT, Spillane C (2009) miR-21 as a key regulator of oncogenic processes. Biochem Soc Trans 37: 918–925.
55. Cheng Y, Zhang C (2010) MicroRNA-21 in cardiovascular disease. J Cardiovasc Transl Res 3: 251–255.
56. Thum T, Gross C, Fiedler J, Fischer T, Kissler S, et al. (2008) MicroRNA-21 contributes to myocardial disease by stimulating MAP kinase signalling in fibroblasts. Nature 456: 980–984.
57. Sayed D, Rane S, Lypowy J, He M, Chen IY, et al. (2008) MicroRNA-21 targets Sprouty2 and promotes cellular outgrowths. Mol Biol Cell 19: 3272–3282.
58. MacLennan DH, Kranias EG (2003) Phospholamban: a crucial regulator of cardiac contractility. Nat Rev Mol Cell Biol 4: 566–577.
